# Biologic Collagen Cylinder with Skate Flap Technique for Nipple Reconstruction

**DOI:** 10.1155/2014/194087

**Published:** 2014-07-10

**Authors:** Brian P. Tierney, Jason P. Hodde, Daniela I. Changkuon

**Affiliations:** ^1^Tierney Plastic Surgery, 2011 Church Street, Suite 805, Nashville, TN 37203, USA; ^2^Cook Biotech Incorporated, 1425 Innovation Place, West Lafayette, IN 47906, USA

## Abstract

A surgical technique using local tissue skate flaps combined with cylinders made from a naturally derived biomaterial has been used effectively for nipple reconstruction. A retrospective review of patients who underwent nipple reconstruction using this technique was performed. Comorbidities and type of breast reconstruction were collected. Outcome evaluation included complications, surgical revisions, and nipple projection. There were 115 skate flap reconstructions performed in 83 patients between July 2009 and January 2013. Patients ranged from 32 to 73 years old. Average body mass index was 28.0. The most common comorbidities were hypertension (39.8%) and smoking (16.9%). After breast reconstruction, 68.7% of the patients underwent chemotherapy and 20.5% underwent radiation. Seventy-one patients had immediate breast reconstruction with expanders and 12 had delayed reconstruction. The only reported complications were extrusions (3.5%). Six nipples (5.2%) in 5 patients required surgical revision due to loss of projection; two patients had minor loss of projection but did not require surgical revision. Nipple projection at time of surgery ranged from 6 to 7 mm and average projection at 6 months was 3–5 mm. A surgical technique for nipple reconstruction using a skate flap with a graft material is described. Complications are infrequent and short-term projection measurements are encouraging.

## 1. Introduction

In 2013, the American Cancer Society estimated that 232,340 new cases of invasive breast cancer would be diagnosed in women. Nipple-areola reconstruction is the last stage in a long and multifaceted journey to restore the presurgical appearance of a person's breast following mastectomy. The presence of a nipple on a reconstructed breast has been shown to be psychologically significant for women who have had mastectomies [1, 2]. Numerous techniques exist for nipple reconstruction, but no method has reliable and consistent aesthetic results [3–13]. Recently, a surgical technique using local tissue flaps combined with cylinders made from a naturally derived biomaterial has been used effectively.

**Figure 1:**
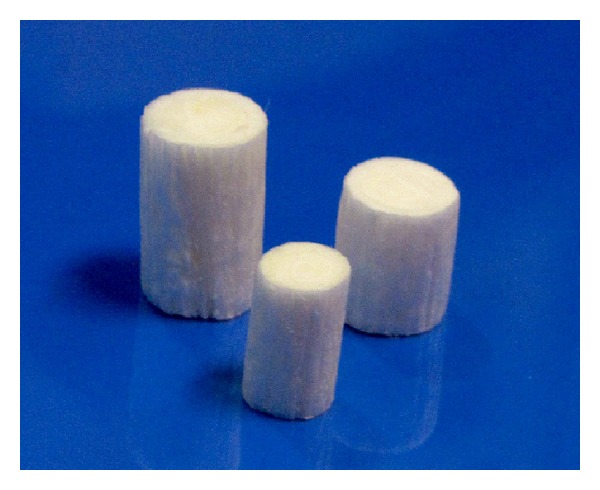
Image of the Biodesign Nipple Reconstruction Cylinder (COOK Inc., Bloomington, IN). Cylinders have a length of either 1.0 cm or 1.5 cm and a diameter of either 0.7 cm or 1.0 cm. All sizes can be trimmed prior to implantation.

The Biodesign Nipple Reconstruction Cylinder (NRC; COOK Inc., Bloomington, IN) is a rolled cylinder of extracellular matrix collagen derived from porcine small intestinal submucosa (SIS) and is intended for implantation to reinforce soft tissue in plastic and reconstructive surgery of the nipple (Figure 1). Like dermis or fascia, SIS is composed of fibrillar collagens and adhesive glycoproteins which serve as a scaffold into which cells can migrate and multiply. Once implanted, the NRC material allows for angiogenesis as well as connective and epithelial tissue growth and differentiation. Furthermore, the material allows cells to migrate into the device and form an organized extracellular matrix (ECM) through the deposition of collagen and other proteins. Over time, this remodeling results in the formation of tissue that is histologically similar to the tissue at the implant site. Eventually, the body metabolizes the device, leaving behind only naturally augmented patient tissue [14–16].

Although some patients decide not to proceed with nipple-areolar reconstruction, the nipple is considered to be a well-defined anatomic marker that contributes significantly to the shape and symmetry of the breast [17]. This retrospective case series describes a skate-flap reconstruction technique in combination with the Biodesign NRC that can be safely performed over breast implants.

## 2. Methods

A retrospective, single-center, single-surgeon, chart review was performed on all postmastectomy breast reconstruction patients who underwent skate-flap nipple reconstruction in combination with a Biodesign NRC between July 2009 and January 2013. Patient demographic data including age, weight, indication for surgery, and cancer stage were collected. Other risk factors, including smoking, preoperative and postoperative chemotherapy, and radiation therapy, were also collected and analyzed. The surgery dates, types of mastectomy, and types of breast reconstruction were recorded for every patient. Outcome evaluations included complications, the need for surgical revision, and nipple projection measurements.

### 2.1. Surgical Technique

**Figure 2:**
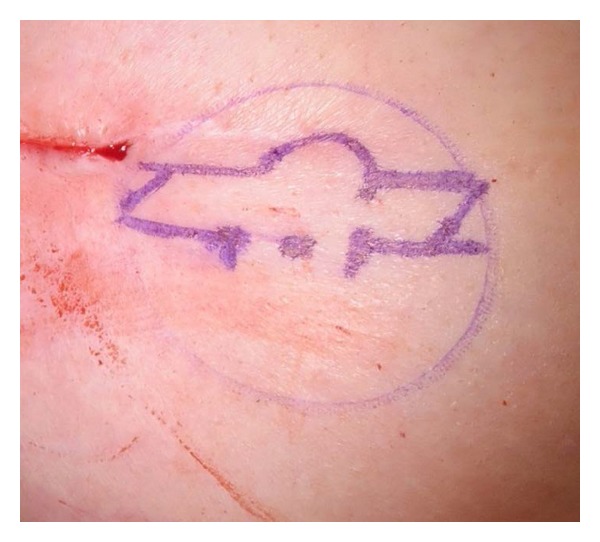
Skate-flap pattern drawn onto the patient's breast to guide the creation of the skin flap.

**Figure 3:**
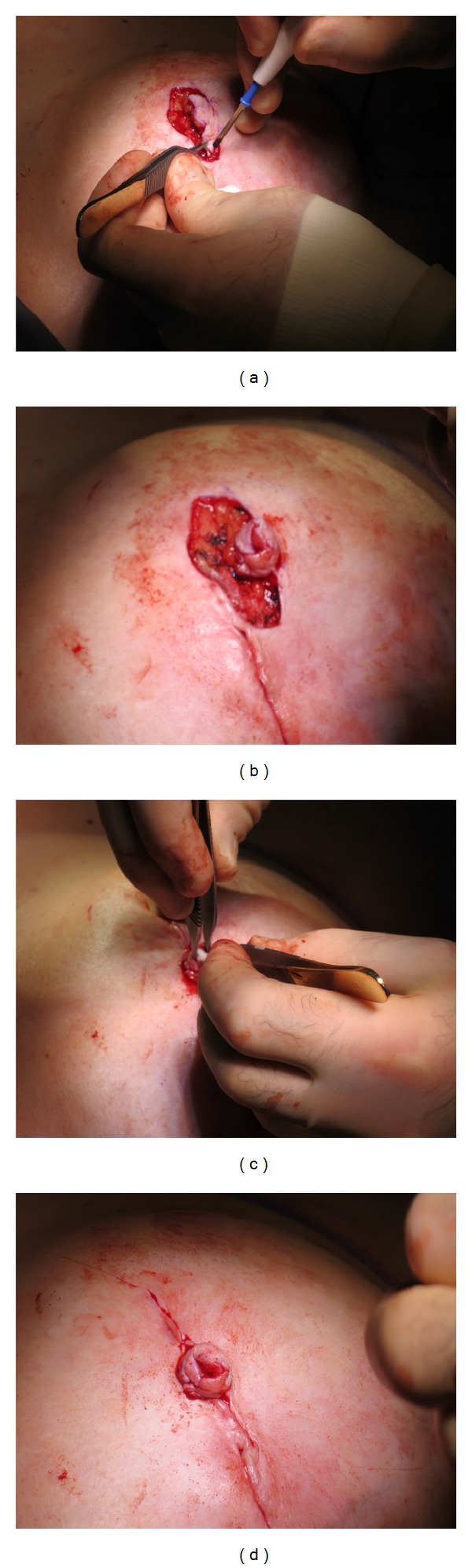
Intraoperative pictures. (a) Skate flap comprised of skin and fatty tissue is cut and lifted along the surgical markings; (b) the ends of the flap are brought together and sutured to allow for cylinder placement; (c) the cylinder is carefully placed inside the flap, (d) resulting in the cylinder being securely wrapped by vascularized skin tissue.

Nipple cylinder diameter (0.7 cm or 1.0 cm) and length (1 cm or 1.5 cm) were selected to closely match the contralateral nipple. If a contralateral nipple was not present, the overall size of the reconstructed breast, the presence or absence of a well-vascularized skin flap, and/or the patient's desired final appearance were considered when determining the cylinder size, allowing for some shrinkage following implant. The position of the nipple was determined with the patient seated in a relaxed position. Using a surgical marker, a skate-flap pattern (Figure 2) was drawn onto the patient's breast to guide the creation of the skin flaps. The NRC was allowed to rehydrate for no greater than 10 seconds before it was placed underneath the appropriate skin flaps, ensuring that an adequate blood supply reached the device. This placement allowed for maximum contact with healthy, well-vascularized tissue and encouraged cell in-growth and tissue remodeling (Figure 3). The cylinder was then secured into place with a combination of 3-0 Vicryl (Ethicon, Somerville, NJ) and 4-0 Monocryl (Ethicon, Somerville, NJ) sutures at the base of the nipple reconstruction to prevent migration of the cylinder into the subcutaneous region beneath the flaps. After reconstruction, incisions were closed with a combination of inverted dermal 3-0 Vicryl sutures and simple interrupted 4-0 Monocryl sutures. The reconstructed nipple was protected using a hard plastic shield, which was left in place for up to 4 weeks. Topical antibiotic cream was not routinely used following surgery, but patients were instructed to use triple antibiotic and return to the clinic if signs of infection were observed when cleansing the area. The areola was later pigmented by tattoo according to standard practice.

## 3. Results

There were 83 women who underwent postmastectomy breast reconstruction and subsequent nipple reconstruction. The average age was 50.4 years (range: 32–73 years) and average body mass index was 28.0 (range: 15.8–48.4). Thirty-three patients (39.8%) had a diagnosis of hypertension, 14 (16.9%) used tobacco products, and 9 (10.8%) had type II diabetes at the time of reconstruction (see Table 1). Indications for mastectomy included infiltrating carcinoma in 40 patients (48.2%), ductal carcinoma* in situ* in 35 patients (42.2%), 1 case (1.2%) each of lobular carcinoma, Paget's disease of the nipple, BRCA+ and high risk benign mass, and 4 patients (4.8%) with unknown indications. Fifteen patients (18.1%) underwent both radiation and chemotherapy, 42 (50.6%) had adjuvant chemotherapy alone, 2 (2.4%) underwent radiation alone, 8 (9.6%) had no adjuvant therapy, and for 16 (19.3%) patients, adjuvant therapy was unknown. Out of the 83 patients, 71 (85.5%) chose to have immediate 2-stage breast reconstruction after mastectomy. The process involved the placement of tissue expanders immediately following mastectomy and a second surgery to replace the expanders with permanent implants. The remaining 12 patients (14.5%) had delayed breast reconstruction, also with tissue expanders.

**Figure 4:**
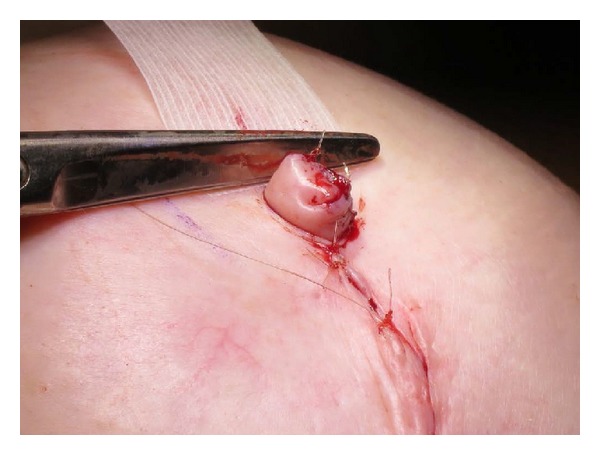
Nipple projection at time of surgery ranged from 6 to 7 mm.

**Figure 5:**
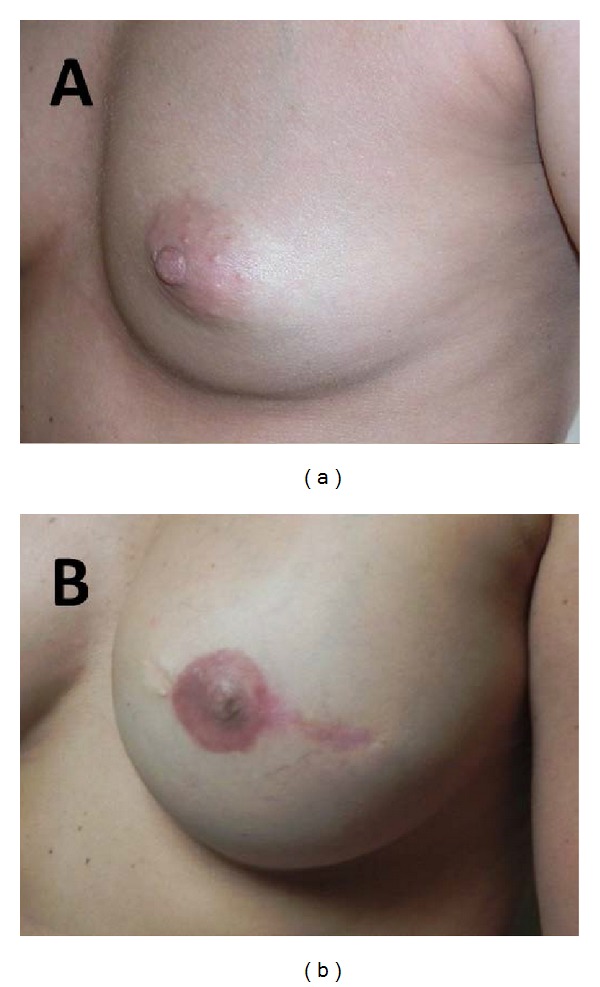
(a) Patient before breast and nipple reconstruction. (b) Same patient after breast and nipple reconstruction.

Using a skate-flap and graft technique in combination with the Biodesign NRC, the total number of nipple reconstructions was 115 (61 unilateral reconstructions and 27 bilateral reconstructions). The only reported complications included 4 cases (3.5%) of NRC extrusion and 5 patients (4 unilateral and 1 bilateral reconstructions, 5.2%) who required surgical revision due to loss of nipple projection (see Table 2). Additionally, two patients reported minor loss of projection but did not require surgical revision. No postoperative cases of infection or allergic reaction to the cylinder were reported. Nipple projection at time of surgery ranged from 6 to 7 mm (see Figure 4) and average projection at 6 months was 3–5 mm. An example of long-term results can be seen in Figure 5.

## 4. Discussion

Published information for nipple-areola reconstruction reveals numerous surgical techniques and variable long-term results in terms of patient satisfaction and sustained nipple projection. Some of the different techniques and local flaps that are used for nipple reconstruction, besides the skate-flap, are the Marshall technique [4], the button-hole technique [5], the C-V flap [6], silicone rods [7], the star-flap [8], the cigar roll flap [9], the hamburger technique [10], the arrow-flap [11], the top-hat-flap [12], the Swiss Roll flap [13], and others. One of the main limitations to this retrospective case review is that it only evaluates the skate-flap technique in combination with the Biodesign NRC; however, this is the senior author's surgical technique of choice and this series demonstrates that the procedure is safe and reliable, with very low complication rates.

Surgeon preference is usually what determines the surgical method of choice for nipple reconstruction. It is difficult to conclude if there is one method that yields superior results than others because very few evidence-based comparisons have been published. It is expected, however, that with every surgical technique for nipple reconstruction that exists today there will be variations in the results, complications, and some degree of projection loss with time. Modifications of current surgical techniques and new device technologies are continuously being developed and tested for lower complication rates and longer lasting nipple projection.

The only cases in this series that required surgical revision were due to loss of nipple projection or cylinder extrusion. The cause of the loss of nipple projection is unknown but may be related to individual patient characteristics or noncompliance in wearing the plastic shield following surgery. Four patients suffered from extrusion of the Biodesign NRC between 2 and 4 weeks after surgery but only 1 patient required further surgical revision. The most likely reason for cylinder extrusion was tension on the sutures that lead to a small degree of tissue ischemia and necrosis of part of the flap. Other risks of nipple reconstruction reported in the literature include localized tip/flap necrosis, partial flap loss, complete flap loss/infection, dehiscence, seroma formation, and an overall expected complication rate of approximately 12% in all patients [17–19]. While all of these risks are applicable when implanting the NRC, this case series did not present any of the aforementioned complications. The limited use of the NRC in patients to date does not allow for an accurate account of the extent of these risks beyond those associated with the surgical procedure alone.

The intended goal of this retrospective case series was to describe a successful skin flap technique and device combination that could be used to reconstruct the nipple-areola complex following the removal and reconstruction of breast tissue. The potential clinical benefits to the subjects are a surgery that can be performed quickly and with minimal morbidity, providing safe, predictable, and long-lasting aesthetic results. Seventy-eight out of 83 patients (94.0%) underwent skate-flap nipple reconstruction in combination with the Biodesign NRC and had no complications. At the time of surgery, average nipple projection ranged from 6 to 7 mm and average projection at 6 months was 3–5 mm, representing a 30%–50% percent loss of projection, which is similar to the projection loss noted following local skin flap nipple reconstruction without graft material augmentation [11]. This loss of projection over time is an important consideration when attempting to decide what size of implant to choose for surgery. It is acknowledged that the relatively short follow-up time of 6 months does not allow us to comment on long-lasting aesthetic outcome at this time.

Alternative treatments to the nipple cylinder for reconstruction include cosmetic tattooing of the areola only, local skin flap nipple reconstruction without graft material augmentation, autologous or composite grafts (i.e., contra lateral nipple, fat grafting, or cartilage) [17, 20, 21], or the use of other biologic materials like human dermis, hyaluronic acid [22], or poly-lactic acid [23].

Currently, more comparative clinical studies are needed to optimize the procedures used to reconstruct the nipple-areola complex following mastectomy. With the use of the Biodesign NRC, there is a potential for reducing patient complications, providing longer-lasting nipple projection and achieving higher patient satisfaction than either autologous graft procedures or flap procedures alone. A combined surgical technique for nipple reconstruction that used a skate flap and an off-the-shelf biologic graft material that resulted in comparable aesthetic results to alternative treatments and promising long-lasting projection was presented. Complications are infrequent and short-term projection measurements are encouraging. Longer-term followup is needed to determine if nipple projection is sustained for longer periods of time and if the added cost of the cylinder is justified by long-term aesthetic outcome.

## Figures and Tables

**Table 1:** Patient demographics.

Total number of patients = 83	
Age	Range: 32–73 years old
	Mean: 50.4 years old

BMI	Range: 15.8–48.4
	Mean: 28.0

Indication for surgery	IC = 40 patients (48.2%)
	DCIS = 35 patients (42.2%)
	LC = 1 patient (1.2%)
	Paget's = 1 patient (1.2%)
	BRCA+ = 1 patient (1.2%)
	Benign mass = 1 patient (1.2%)
	Unknown = 4 patients (4.8%)

Diabetes (Type II)	9 patients (10.8%)

Hypertension	33 patients (39.8%)

Smoking (or tobacco products)	14 patients (16.9%)

Preoperative chemotherapy	22 patients (26.5%)

Postoperative chemotherapy	48 patients (57.8%)

Chemotherapy alone (no radiation)	42 patients (50.6%)

Preoperative radiation	6 patients (7.2%)

Postoperative radiation	14 patients (16.9%)

Radiation alone (no chemotherapy)	2 patients (2.4%)

Combined chemotherapy and radiation	15 patients (18.1%)

No adjuvant cancer treatment	8 patients (9.6%)

Unknown cancer treatment	16 patients (19.3%)

IC: infiltrating carcinoma, DCIS: ductal carcinoma in situ, LC: lobular carcinoma, Paget's: Paget's disease of the nipple, and BRCA+: positive breast cancer gene.

**Table 2:** Nipple reconstruction complications.

Patient	Date of nipple reconstruction	Surgery procedure used	NRC: L, R, or bilateral	Complications
1	7/10/2009	Skate + NRC	Left	Lost projection, surgical revision required
	10/16/2009	Revision with second NRC		

2	1/19/2011	Skate + NRC	Bilateral	Lost projection, surgical revision required
	4/5/2011	Revision with second NRC		

3	4/12/2011	Skate + NRC	Right	Cylinder extrusion 3 weeks post-op; projection still viable, no surgical revision required

4	3/28/2011	Skate + NRC	Right	Patient displeased with projection, surgical revision required
	7/26/2011	Revision with second NRC		

5	5/18/2011	Skate + NRC	Left	Cylinder extrusion 2 weeks post-op; projection still viable, no surgical revision required

6	2/7/2012	Revision with NRC	Right	Loss of projection due to radiation therapy; cylinder extrusion 3 weeks post-op; projection still viable, no surgical revision required

7	9/30/2011	Skate + NRC	Right	Lost projection, surgical revision required
	10/26/2012	Revision with second NRC		

8	10/5/2011	Skate + NRC	Left	Cylinder extrusion 4 weeks post-op; lost projection, surgical revision required
	3/9/2012	Revision with second NRC		

9	10/14/2012	Skate + NRC	Left	Lost projection, surgical revision required
	2/24/2012	Revision with second NRC		

Skate: skate-flap nipple reconstruction technique and NRC: Biodesign Nipple Reconstruction Cylinder.
